# The efficacies of modified mechanical post conditioning on myocardial protection for patients undergoing coronary artery bypass grafting

**DOI:** 10.1186/1749-8090-7-73

**Published:** 2012-08-09

**Authors:** Serkan Durdu, Mustafa Sirlak, Demir Cetintas, Mustafa Bahadir Inan, Sadik Eryılmaz, Evren Ozcinar, Levent Yazicioglu, Atilla Halil Elhan, Ahmet Ruchan Akar, Adnan Uysalel

**Affiliations:** 1 Department of Cardiovascular Surgery, Heart Center, Ankara University School of Medicine, Mamak Street, 06340, Dikimevi-Ankara, Turkey; 2 Stem Cell Institute, Ankara University, Ankara, Turkey; 3 Department of Biostatistics, Ankara University School of Medicine, Ankara, Turkey

**Keywords:** Cardiopulmonary bypass, Myocardial protection, Ischemia-reperfusion injury, Coronary artery bypass grafting, Post-conditioning

## Abstract

**Background:**

Coronary artery bypass grafting (CABG) with cardioplegic cardiac arrest and cardiopulmonary bypass (CPB) is associated with myocardial injury. The aim of this study was to investigate whether a modified mechanical post-conditioning (MMPOC) technique has a myocardial protective effect by enhancing early metabolic recovery of the heart following revascularization.

**Methods:**

A prospective, randomized trial was conducted at a single-center university hospital performing adult cardiac surgery. Seventy-nine adult patients undergoing first-time elective isolated multivessel coronary artery bypass grafting were prospectively randomized to MMPOC or control group. Anesthetic, cardiopulmonary bypass, myocardial protection, and surgical techniques were standardized. The post reperfusion cardiac indices, inotrope use and biochemical-electrocardiographic evidence of myocardial injury were recorded. The incidence of postoperative complications was recorded prospectively.

**Results:**

Operative characteristics, including CPB and aortic cross-clamp time, were similar between the two groups (p>0.05). The MMPOC group had lower troponin I and other cardiac biomarkers level post CPB and postoperatively, with greater improvement in cardiac indices (p<0.001). MMPOC shortened post surgery hospitalization from 9.1 ± 2.1 to 7.5 ± 1.6 days (p<0.001).

**Conclusions:**

MMPOC technique promotes early metabolic recovery of the heart during elective CABG, leading to better myocardial protection and functional recovery.

## Background

Reperfusion has the potential to cause additional reversible and irreversible damage to the myocardium, which is called reperfusion injury [1,2]. The existence of post-conditioning (POC) is the newest evidence that has emerged to support the concept of reperfusion injury. The term post conditioning refers to the phenomenon in which multiple brief periods of reperfusion interspersed with brief periods of ischemia (10–60 s) result in a reduction in infarct size [3,4]. Generally, three cycles of ischemia/reperfusion are required to produce a maximal POC effect, although four and six cycles have been shown to be effective by some investigators [3,4]. However, it is the interval of reperfusion and ischemia that is the most critical factor in determining how efficacious POC will be. POC protocols shown to be maximally effective at reducing infarct size range from 10 to 60 s depending on the specific species being studied [3,4].

The aim of our study was to determine the efficacy of a modified type of mechanical post-conditioning (MMPOC) in patients undergoing elective coronary revascularization, with specific attention to biochemical markers of ischemic injury and post-surgical recovery of the patients and to show whether there is room for protection by post-conditioning amongst all the other cardioprotective factors.

## Methods

### Patients and protocol

This study was approved by the Institutional Review Board of the University of Ankara and consisted of 79 patients undergoing elective primary coronary revascularization with ≥99% stenosis of the left anterior descending (LAD) artery. So as to include patients with relatively large volumes of at-risk myocardium, we limited our analysis to those patients exhibiting proximal occlusion of the LAD. Patients undergoing valve replacement, combined valve replacement/coronary revascularization, or preoperative coronary revascularization was excluded from the study. Informed consent was obtained from all patients before enrollment.

### Randomisation

Patients were allocated to the MMPOC group or the study group using a computer-generated randomization code. Participants were randomly assigned in a 1:1 ratio. The study was open label and the primary investigator, who was not the treating physician and nurses informed the participants about their allocated treatment. Data for primary outcomes were assessed by use of a computer.

### Data collection and definitions

Baseline, procedural, and follow-up data were stored prospectively in a database located at the University of Ankara. Patients’ preoperative risk factors were recorded and EuroSCOREs were calculated for each patient. Patients’ preoperative characteristics were recorded including age, sex, size, preexisting medical conditions, preoperative medications, preoperative ejection fractions. Intraoperative variables of which number of coronary bypass grafts, duration of cardiopulmonary bypass (CPB), duration of aortic cross-clamp, requirement for inotropic drugs, and/or intra-aortic balloon support, and blood product use were included. Postoperative data comprised myocardial infarction, cardiac tamponade, reoperation for occlusion or other causes, requirement of intra-aortic balloon pump support, neurologic complications, renal dysfunction, chest tube drainage during the first 24 postoperative hours, total chest tube drainage, the length of mechanical ventilator support, pneumonia, multiorgan failure, gastrointestinal complications, sepsis, coma, the length of intensive care unit (ICU) stay, and readmission within 90 days after surgery.

Adverse events were defined as death, perioperative myocardial infarction, stroke, re-exploration due to bleeding, respiratory insufficiency, and renal failure. Perioperative myocardial infarction (MI) was defined as either new Q waves or ischemic ST segment changes with concomitant elevations of creatine kinase isoenzyme (CK-MB) > 5 times the upper limit of the reference range or a CK-MB to total creatine kinase (CK) ratio > 10% occurring within 48 hours after surgery or troponin I (TnI) > 1 ng/mL. Renal dysfunction was defined as rise of serum creatinine above 2.5 mg/dL and/or a need for hemodialysis. The surgical team examined all patients about 4-6 weeks after discharge and annually thereafter for two years.

### Anesthetic and surgical considerations

Anesthesia was maintained with isoflurane. Hypertension was treated by increasing the concentration of isoflurane or by the administration of nitroglycerin if increasing the depth of anesthesia was ineffective. Hypotension was corrected using volume replacement or phenylephrine, as clinically indicated. An additional dose of 5 mg of midazolam was provided during rewarming from CPB. Inotropic agents (dobutamine 5 μg/kg/min) were initiated for a cardiac indices (CI) <2.0 L/min/m^2^ after separation from CPB. At sternal closure, an infusion of propofol was started (25-75 μg/kg/min), and the isoflurane was discontinued. Propofol sedation was continued in the ICU until weaning of ventilatory support was initiated.

**Figure 1:**
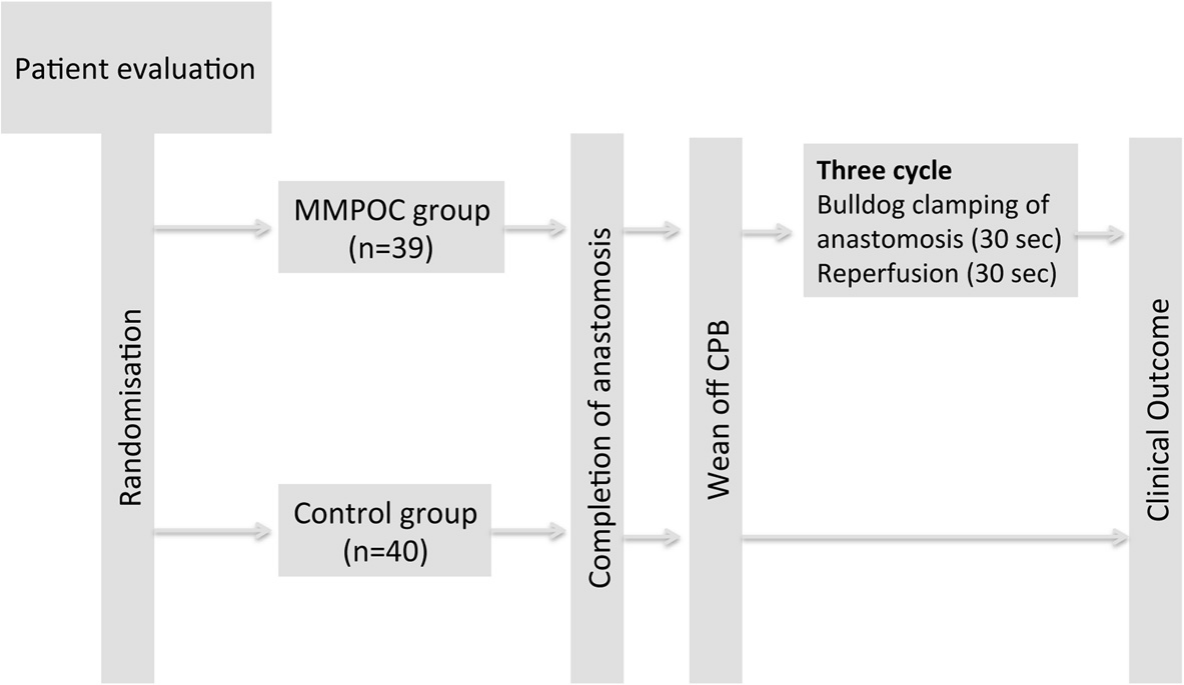
**The Modified Mechanical Postconditioning (MMPOC) Protocol.** Patients were allocated to the MMPOC group (n = 39) or the Control group (n = 40) using a computer-generated randomization code. During MMPOC protocol, proximal bypass anastomosis were completed and after wean off CPB phase, three cycle of bulldog clamping of anastomosis for 30 seconds and anastomosis were reperfused for 30 seconds. Then, clinical outcomes of the patients were recorded.

All patients had coronary artery bypass grafting (CABG) with the use of CPB, which was conducted with a roller pump and a membrane oxygenator primed with a solution consisting of 1 L of Ringer’s lactate, 5000 IU of heparin, 750 mL of Pentaspan (DuPont Pharmaceuticals Co, Newark, DE), and 44 mEq of bicarbonate. During CPB, pump flow was set at 2.4 times the body surface area, and mean arterial pressure maintained between 50 and 60 mm Hg. The temperature was allowed to drift with active rewarming at the end of CPB. Cardioplegia solution (Plegisol, Hospira,Inc, Lake Forest, IL), which was used at the discretion of the cardiac surgeon, was free of glucose and consisted of high-dose (100 mEq/L) and low-dose (40 mEq/L) potassium. Cardioplegia was given in an anterograde fashion with blood in a ratio of 1:4. Blood cardioplegia was also administered with each successive distal vein graft anastomosis. The proximal vein graft anastomosis were performed after the distal ones under tangential aortic clamp. The left internal thoracic artery was used in all cases to bypass the LAD, while other coronary arteries received saphenous vein grafts. In patients with severe aortic disease where intermittent cross-clamping may be harmful, we used the MMPOC technique immediately after completion of the proximal anastomosis and weaning off CPB, in the MMPOC group we applied bulldog clamps to the grafts anatomized to the coronary arteries as in a similar manner and duration explained by Zhao et al before 2 [4]. Our MMPOC technique consisted of applying three 30-sec alternate episodes of occlusion with bulldog clamps and releasing of the bypassed grafts providing reperfusion. This method was applied immediately after CPB and in this way uncontrolled reperfusion was disallowed in the study group (Figure 1). This method was applied immediately after CPB and in this way uncontrolled reperfusion wasn’t permitted in the study group.

### Functional recovery and postoperative cardiac biomarkers

The CI, hemodynamic parameters and cardiac biomarkers were evaluated in 9 different time points (immediately after the induction of anesthesia; following completion of the proximal anastomosis; immediately off CPB; immediately after MMPOC; 2, 6, 12, 24, and 48 hours-post MMPOC). The CI was calculated to determine the change in the CI as an indicator of functional recovery. Central venous pressure (CVP), mean pulmonary artery pressure (MPAP) and pulmonary capillary wedge pressure (PCWP) measurements were compared at the same time points to ensure similar preload filling status.

The baseline values of serum CK-MB, and TnI concentration were taken as described above. All the TnI samples were stored deep-frozen for quantitative analysis. TnI was analyzed using an AxSYM TnI Reagent Kit as a micro particle enzyme immunoassay method and an AxZYM analyzer (Abbott Laboratories, Abbot Park, IL). The upper reference limit was 0.50 g/L, and the cutoff limit for diagnosing acute myocardial infarction was 1.00 [g/L].

### Sample size and randomisation

A repeated measures design has MMPOC and control groups of 39 and 40 subjects respectively for a total of 79 patients. Each subject is measured 6 times. The between-subject standard deviation is 0.41 and the within-subject standard deviation is 1.00. This design achieves 96% power when an F test is used to test the group-factor at a 5% significance level and the actual standard deviation among the appropriate means is 0.17 (an effect size of 0.42), achieves 91% power when an F test is used to test the time-factor at a 5% significance level and the actual standard deviation among the appropriate means is 0.19 (an effect size of 0.19), and achieves 83% power when an F test is used to test the (group X time) interaction at a 5% significance level and the actual standard deviation among the appropriate means is 0.17 (an effect size of 0.17) [5].

### Statistical analysis

Nominal variables were assessed by Chi square test or Fisher’s Exact test, where applicable. Continuous and ordinal variables were evaluated between two groups by Student’s *t* test and Mann–Whitney *U* test, respectively. Two-way analysis of variance (ANOVA) for repeated measures was used to assess the main effect of time as within-factor and post conditioning versus control as grouping factor and interactions between them (group X time). If a significant effect was detected, pair wise comparisons were evaluated using the Bonferroni test. A *p* value less than 0.05 was considered significant. All analyses were performed using SPSS® Statistics 18.0 (SPSS Inc, Chicago, Illinois, USA).

## Results

**Figure 2:**
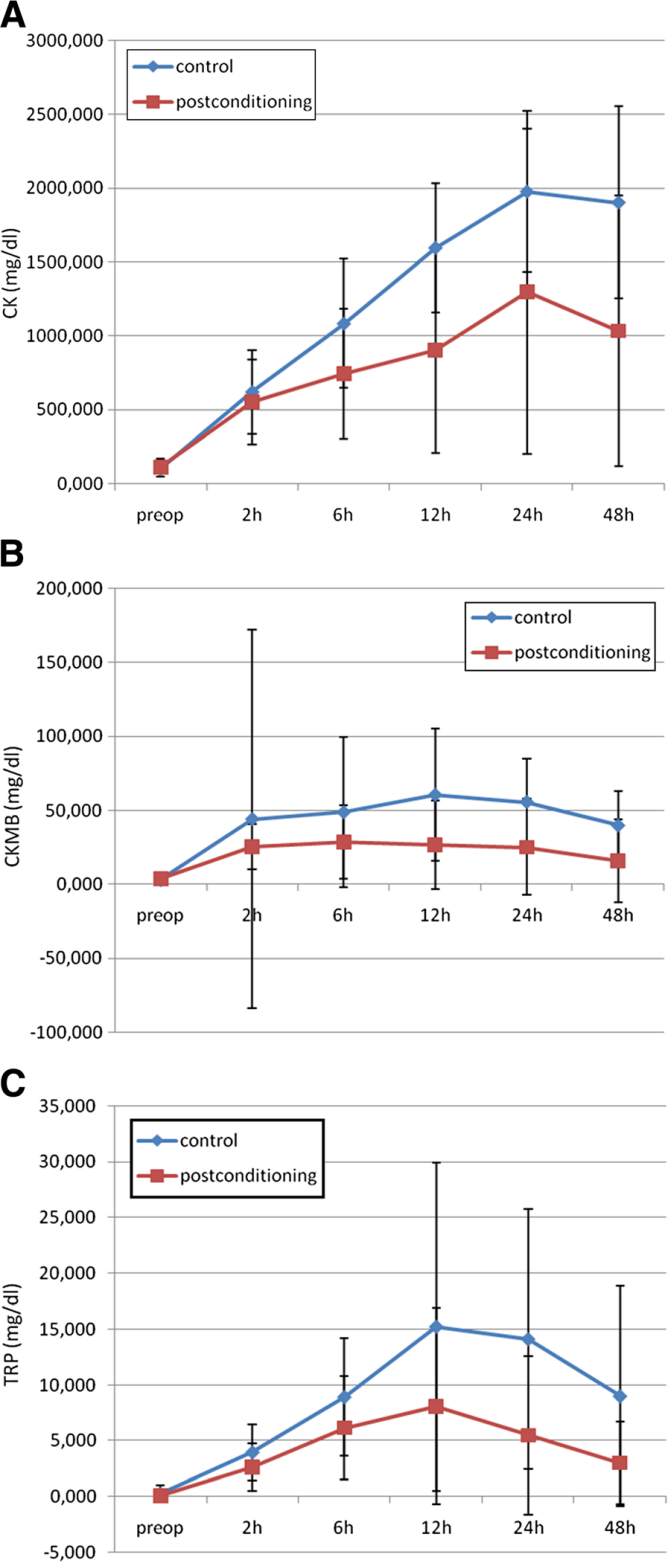
**The Kinetics of Cardiac Biomarkers.A**; Time Course of Creatine Kinase (CK). Preoperative CK values were statistically insignificant for the control and postconditioning groups. Postoperative 2 hour point, CK values were similar. After 6 hour, CK levels for the MMPOC group and Control group began to depart and this significant difference continued to the 12 hour, 24 hour and 48 hour after the procedure. **B**; Time Course of Creatine Kinase Myocard-Brain (CK-MB). Preoperative CK-MB values were statistically insignificant for the control and postconditioning groups. Postoperative 2 hour point, CK-MB values for the two groups were began to differentiated statistically. CK-MB levels for the MMPOC and Control group were statistically significant for the 6 hour, 12 hour, 24 hour, and 48 hour time points. **C**; Time Course of Troponin-I (TnI). Preoperative TnI values were statistically insignificant for the control and postconditioning groups. Postoperative 2 hour point, TnI values were similar. After 6 hour, TnI levels for the MMPOC group and Control group began to depart and this significant difference continued to the 12 hour, 24 hour and 48 hour after the procedure. *Blue line: Control, Red line: MMPOC*.

The patient characteristics as well as preoperative cardiac biomarkers and hemodynamic variables were similar within each study group (p>0.05) (Table 1 and Figure 2 A,B,C). The kinetics of cardiac biomarkers followed almost the same route and remained lower in the study group throughout the study period. But statistically significant differences occurred in different time points. CK and CK-MB remained lower in the study group from the 6^th^ and 12^th^ post-conditioning hours till the end of the study respectively in the MMPOC group (p<0.001). Although TnI levels remained lower in the study group, the values reached statistical difference after the 24^th^ hours (p<0.001) (Figure 2 A,B,C).

**Table 1:** Patient Characteristics

		**MMPOC (n, 39)**	**Control (n, 40)**	**P Value**
Age (Yr)	Mean ± SD	61.6 ± 8.1	62.4 ± 7.1	0.833
	Median (Min-Max)	62 (45-73)	63 (48-77)	
NHYA	Mean ± SD	1.87 ± 0.76	1.72 ± 0.67	0.431
	Median (Min-Max)	2 (1-4)	2 (1-3)	
LVEF (%)	Mean ± SD	47.6 ± 5.5	46.1 ± 5.7	0.185
	Median (Min-Max)	48 (38-57)	44.5 (39-57)	
EuroSCORE	Mean ± SD	3.05 ± 1.37	2.67 ± 1.42	0.193
	Median (Min-Max)	3 (1-6)	2 (1-6)	
LMCA disease	n (%)	3 (7.7)	2 (5.0)	0.675
History of Hypertension	n (%)	15 (38.5)	17 (42.5)	0.715
Diabetes Mellitus	n (%)	15 (38.5)	17 (42.5%)	0.715
Hb (g/dl)	Mean ± SD	12.1 ± 0.46	12.3 ± 0.57	0.358
	Median (Min-Max)	12.3 (10.6-13.1)	12.3 (11.8-14.5)	
Glucose (mg/dl)	Mean ± SD	101.4 ± 14.8	98 ± 12.1	0.376
	Median (Min-Max)	98 (75-131)	95 (75-125)	
Creatinine (mg/dl)	Mean ± SD	1.13 ± 0.19	1.15 ± 0.15	0.484
	Median (Min-Max)	1.1 (0.8-1.8)	1.2 (0.9-1.4)	
BUN (mg/dl)	Mean ± SD	25.6 ± 4.9	26.5 ± 5.1	0.421
	Median (Min-Max)	25 (14-36)	25 (20-34)	
Protein (mg/dl)	Mean ± SD	6.60 ± 0.19	6.61 ± 0.22	0.841
	Median (Min-Max)	6.6 (6.1-7.0)	6.7 (6.2-7.2)	
Albumin (mg/dl)	Mean ± SD	3.50 ± 0.20	3.46 ± 0.13	0.277
	Median (Min-Max)	3.5 (3.0-3.9)	3.4 (3.3-3.8)	
INR	Mean ± SD	1.05 ± 0.07	1.03 ± 0.07	0.450
	Median (Min-Max)	1.02 (0.9-1.3)	1.02 (0.8-1.2)	

*SD* = standard deviation, *Min* = minimum, *Max*: maximum, *MMPOC* = modified mechanical post-conditioning *NYHA*, New York Heart Association, *LVEF* = left ventricular ejection fraction, *LMCA* = left main coronary artery, *Hb* = hemoglobin, *BUN*: blood urea nitrogen, *INR*: international normalized ratio.

Nominal variables were assessed by Chi square test or Fisher’s Exact test, where applicable. Continuous and ordinal variables were evaluated between two groups by Student’s *t* test and Mann–Whitney *U* test, respectively.

### Intraoperative characteristics

**Figure 3:**
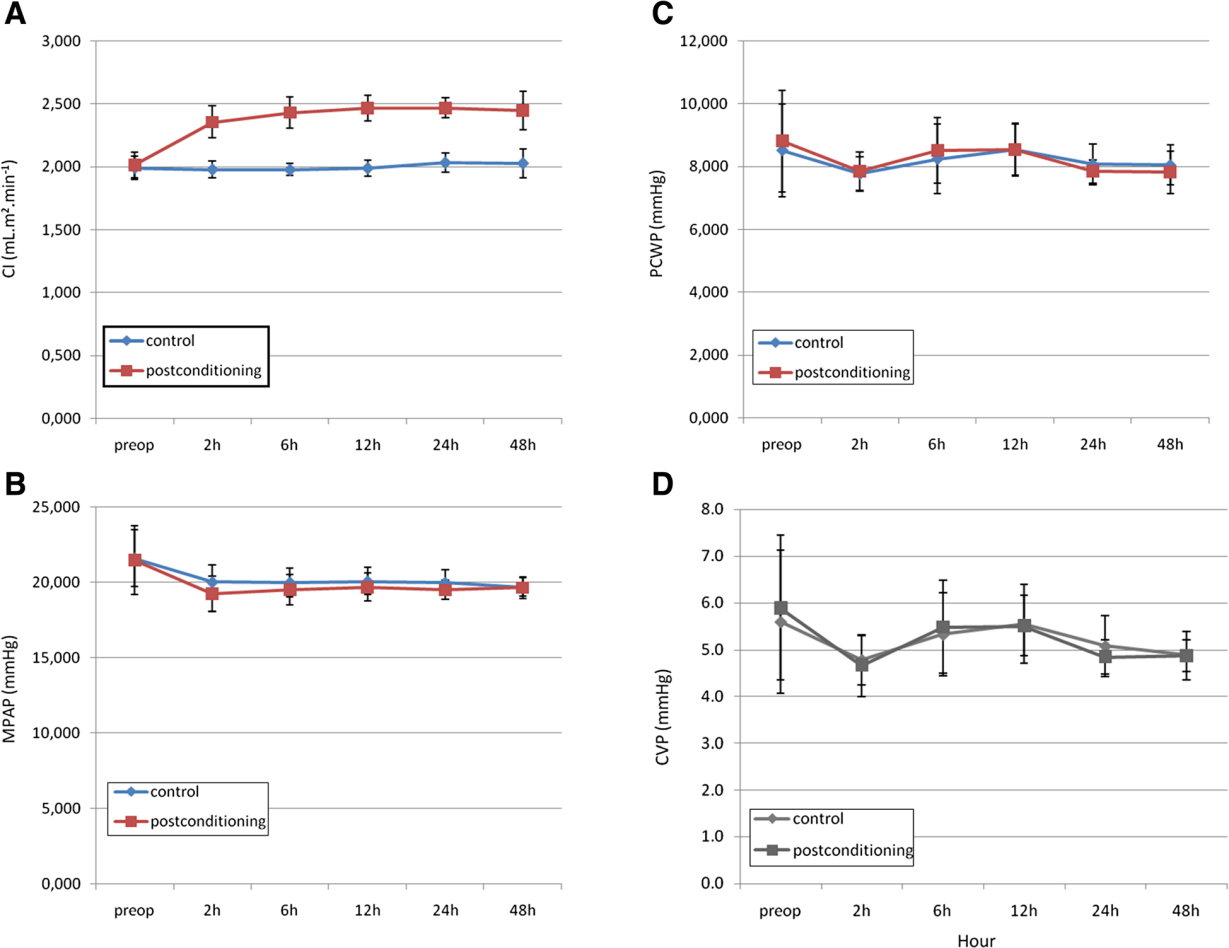
**Patients’ Hemodynamic Data.A**; Time Course of Cardiac Index (CI). Effect of MMPOC on recovery of CI after 2 hours. Preoperative CI values were statistically insignificant for the control and postconditioning groups. Postoperative 2 hour point, CI values for MMPOC group exerted significant difference. After 2 hour point, MMPOC group patients’ CI values showed significant improvement. **B**; Time Course of Mean Pulmonary Arterial Pressure (MPAP). MPAP values for both groups were statistically insignificant from the beginning of the procedure. **C**; Time Course of Pulmonary Capillary Wedge Pressure (PCWP). PCWP values for both groups were statistically insignificant from the beginning of the procedure. **D**; Time Course of Central Venous Pressure (CVP). CVP values for both groups were statistically insignificant from the beginning of the procedure. *Blue line: Control, Red line: MMPOC*.

The CPB time was not statistically different between the groups (p *=* 0.062) nor was the aortic cross-clamp time (p = 0.082). The mean number of grafts was similar between the groups (p = 0.974). The operative time was shorter in the MMPOC group (Table 2), however it was statistically insignificant (p = 0.619). This period also include the post conditioning time. CI were comparable between the two groups in the first period (2.01 ± 0.10 L/min/m^2^ in MMPOC group versus 1.99 ± 0.09 L/min/m^2^ in control group, p = 0.362). Under the similar preload filling status, patients in MMPOC group had higher post-conditioning CI in all time points (p<0.001). The actual values of PCWP, MPAP and CVP remained similar between the two groups in all time points (p>0.05) (Figure 3 A,B,C,D).

**Table 2:** Intraoperative characteristics

		**MMPOC(n = 39)**	**Control(n = 40)**	***P value***
Operation Time (min)	Mean ± SD	154.3 ± 17.0	158.3 ± 22.6	0.619
CPB Time (min)	Mean ± SD	74.1 ± 27.3	81.7 ± 17.0	0.062
Cross Clamp Time (min)	Mean ± SD	41.2 ± 14.9	44.7 ± 8.9	0.082
IABP	n (%)	0 (0)	2 (5)	0.241
No. of graft/patient		2.92 ± 0.83	2.87 ± 0.64	0.974

*MMPOC* = modified mechanical post-conditioning, *SD* = standard deviation, *CPB*, cardio-pulmonary bypass, *IABP*, intra aortic balloon pump.

Nominal variables were assessed by Chi square test or Fisher’s Exact test, where applicable. Continuous and ordinal variables were evaluated between two groups by Student’s *t* test and Mann–Whitney *U* test, respectively.

### Postoperative characteristics

The median length of intubation was similar between the groups (*p =* 0.818). The MMPOC group patients had lower length of stay in the ICU and in the hospital compared to the control group (p<0.001). Importantly, use of MMPOC shortened postoperative hospitalization by more than one day. Postoperative complications were similar in both groups. There were three and five perioperative myocardial infarctions in MMPOC and control groups respectively (p = 0.712). While two hospital deaths were seen in control group, none of the patients died in MMPOC group (p = 0.499). Wound infection rates, amount of bleeding and postoperative atrial fibrillation incidences were similar between the groups as shown in Table 3. The improvement of left ventricular function in the MMPOC group tended to decrease requirements for pharmacologic inotropic support (p = 0.004).

**Table 3:** Postoperative characteristics

		**MMPOC (n = 39)**	**Control (n = 40)**	**P Value**
Postoperative inotropes	n (%)	19 (48)	32 (80)	0.004
IABP	n (%)	2 (5)	4 (10)	0.675
Bleeding (ml)	Mean ± SD	569.2 ± 120.5	545.6 ± 97.1	0.214
Mechanical Ventilation (hrs)	Mean ± SD	12.0 ± 3.22	15.6 ± 3.21	0.818
ICU (hrs)	Mean ± SD	15.8 ± 2.3	18.8 ± 3.7	0.000
Postoperative MI	n (%)	3 (7.7)	5 (12.5)	0.712
Hospitalization (days)	Mean ± SD	7.5 ± 1.6	9.1 ± 2.1	0.001
Wound Infection	n (%)	4 (10)	2 (5)	0.432
Atrial Fibrillation	n (%)	6 (15)	5 (12.5)	0.711
In-hospital mortality	n (%)	0 (0)	2 (5.0)	0.499

*MMPOC* = modified mechanical post-conditioning, *IABP* = intra aortic balloon pump, *ICU* = intensive care unit, *SD* = Standard Deviation.

Nominal variables were assessed by Chi square test or Fisher’s Exact test, where applicable. Continuous and ordinal variables were evaluated between two groups by Student’s *t* test and Mann–Whitney *U* test, respectively.

## Discussion

This study demonstrates that MMPOC is associated with increased CI and less cardiomyocyte injury. These findings are important because previous studies have demonstrated that low cardiac output, the need for inotropic support, and biochemical evidence of myocyte injury are important risk factors for prolonged ventilation, ICU stay, and early mortality after CABG [6]. The requirements for pharmacologic inotropic support during the post bypass period reached to statistical significance between the groups. Importantly, use of MMPOC shortened postoperative hospitalization by more than one day.

In our study, we hypothesized that post conditioning would provide protection against myocardial ischemia-reperfusion in adult patients undergoing CPB for CABG and therefore conducted a controlled trial to evaluate the effect of post conditioning on myocardial protection. We applied a new method for the similar hypothesis set up in the other post-conditioning studies [7-11]. Our study setting was different compared to the familiar studies especially for the initiation of the post-conditioning protocols. Our method started in the time of weaning from CPB while all the remaining studies started after aortic declamping. Although larger controlled clinical trials are needed to determine the effect of ischemic post conditioning on the reduction of myocardial necrosis in cardiac surgery, the results of the study conducted by Luo et al. Provided support for ischemic post conditioning as an adjunct to cardioplegia in reduction of myocardial necrosis in adult cardiac surgery [7]. Parallel to the aforementioned studies and statements, our study investigated the cardioprotective efficacy of MMPOC in patients undergoing elective coronary revascularization. The current study focused on cardiac biomarker release after CABG surgery. Application of MMPOC prevented the post-bypass increases in TnI, CK and CK-MB.

In our study, to overcome the problem of predicting the volume of at-risk myocardium or collateral perfusion into the ischemic area, we planned to surmount this potentially confounding factor by including only those patients exhibiting proximal occlusion of the LAD artery with or without occlusions of the other coronary arteries as indicated by Darling et al [12]. It can be hypothesized that the better preservation of cardiac function obtained by MMPOC evidenced by data on reduced postoperative TnI release and the reduced need for inotropic support resulted in improved global tissue perfusion with better recovery from surgery as shown by shorter postoperative hospitalization. The mechanisms underlying the benefits of MMPOC are similar to other mechanical POC techniques described before with the advantage of not requiring repetitive aortic cross-clamping.

Post-conditioning as with ischemic preconditioning has the drawback of intermittent cross-clamping which prompted us to modificate the original technique. Unlike pre-conditioning, post-conditioning does not require initiation prior to the ischemic event. This aspect offers several interesting opportunities to the cardiac surgeon. Post-conditioning can be used in situations where preconditioning is difficult or impossible to achieve. The cardiac transplant surgeons can also benefit from post-conditioning. The storage time of the cardiac allograft can often exceed the protective effects of early pre-conditioning and therefore, post-conditioning may play an important role for allograft protection following storage and transplantation. Remote post-conditioning has also been demonstrated in an animal model [13]. Kerendi et al. demonstrated in a rodent model that remote post-conditioning using renal artery occlusion reduced infarct size by 50% [13]. Ischemic post-conditioning requires intermittent cross-clamping of another organ to protect the myocardium which may be associated with morbidity or increased complexity and length of the operative procedure. The optimal post-conditioning strategy would be pharmacologic. In this respect a variety of diverse pharmacological post-conditioning agents including inhalational anesthetics, G-protein coupled receptor ligands such as opioids, adenosine and bradykinin, growth factors such as insulin and erythropoietin, natriuretic peptides, adipocytokines, and statins have been linked to the activation of the reperfusion injury salvage kinase pathway, a critical component of this signaling pathway [14,15].

Pharmacological post-conditioning would avoid the adverse consequences associated with intermittent cross-clamping and provide a simple method of myocardial protection following all cardiac procedures. A potential drawback of post conditioning is that it offers protection after the ischemic insult has occurred and by then, the myocardial injury may be so extensive that post conditioning is of little benefit [13].

The heightened sensitivity and specificity of TnI and of CK-MB have rendered such markers ideal in revealing a cardiac injury following an ischemic insult of a different nature. Intraoperative TnI release has a functional significance because it is closely related to ischemic time and reflects delayed recovery of left ventricular function and oxidative metabolism; therefore, its measurement can be used as an indicator of myocardial injury sustained during CABG [16]. A recent study by Luo et. al showed the postoperative release of CK-MB was significantly decreased in POC patients undergoing valve replacement [7]. Moreover, in another study, the same group showed the beneficial effects of POC in a patient subgroup undergoing surgery for tetralogy of Fallot [8]. The major finding of that study is that POC reduced postoperative peak release by 34% for CK-MB and 50% for TnI, respectively, suggesting that POC reduced myocardial injury. Another study conducted by Kin et. al demonstrated that pharmacologic post conditioning with adenosine administered through an arterial catheter during CPB produces protective effects against myocardial reperfusion injury in cardiac operations [17]. A possible mechanism of protection, namely, prevention of endothelial ischemia-reperfusion injury by POC was shown by recent studies [18,19].

## Conclusion

We suggest that these cardioprotective effects of MMPOC could have clinical implication in terms of clinical outcome (reduced postoperative length of stay) in CABG operations. Significantly shorter hospital stays will certainly result in lower total hospital costs and nosocomial infections. It can be an adjunct to the other regimes of cardioprotection. Furthermore, our study includes moderately high risk patients with impaired left ventricular ejection faction, medium EuroSCORE and high New York Heart Association (NYHA) status. We assume that improvement in clinical outcome after CABG for patients treated with MMPOC could be demonstrated in a larger study involving patents with worse left ventricular function.

Briefly, our MMPOC technique adjusted after CPB promotes early metabolic recovery of the heart during elective CABG and leads to better myocardial protection and functional recovery.

## Abbreviations

POC: Post-conditioning; MMPOC: Modified type of mechanical post-conditioning; LAD: Left anterior descending; CPB: Cardiopulmonary bypass; ICU: Intensive care unit; MI: Myocardial infarction; CK-MB: Creatine kinase isoenzyme; CK: Creatine kinase; TnI: Troponin I; CI: Cardiac indices; CABG: Coronary artery bypass grafting; CVP: Central venous pressure; MPAP: Mean pulmonary artery pressure; PCWP: Pulmonary capillary wedge pressure; NYHA: New York Heart Association.

## Competing interests

There is no undisclosed ethical problem or competing interests related to the submitted manuscript.

## Authors’ contributions

SD, MS, DC participated in the design of the study and drafted the manuscript. EO, MBI, ARA, LY, SE, AU participated in performing trial patients and data collection. AHE, DC participated in the statistical analysis. SD, MS conceived of the study, participated in its design and coordination, helped to draft the manuscript and give final approval of the version to be published. All authors read and approved the final manuscript.
